# Recent Advances on Macrocyclic Trichothecenes, Their Bioactivities and Biosynthetic Pathway

**DOI:** 10.3390/toxins12060417

**Published:** 2020-06-23

**Authors:** Muzi Zhu, Youfei Cen, Wei Ye, Saini Li, Weimin Zhang

**Affiliations:** Guangdong Provincial Key Laboratory of Microbial Culture Collection and Application, State Key Laboratory of Applied Microbiology Southern China, Guangdong Institute of Microbiology, Guangdong Academy of Sciences, Guangzhou 510070, China; zhumz@gdim.cn (M.Z.); cenyoufei@163.com (Y.C.); yewei@gdim.cn (W.Y.); lisn@gdim.cn (S.L.)

**Keywords:** macrocyclic trichothecenes, bioactivities, putative biosynthetic pathway, macrocycle formation

## Abstract

Macrocyclic trichothecenes are an important group of trichothecenes bearing a large ring. Despite the fact that many of trichothecenes are of concern in agriculture, food contamination, health care and building protection, the macrocyclic ones are becoming the research hotspot because of their diversity in structure and biologic activity. Several researchers have declared that macrocyclic trichothecenes have great potential to be developed as antitumor agents, due to the plenty of their compounds and bioactivities. In this review we summarize the newly discovered macrocyclic trichothecenes and their bioactivities over the last decade, as well as identifications of genes *tri17* and *tri18* involved in the trichothecene biosynthesis and putative biosynthetic pathway. According to the search results in database and phylogenetic trees generated in the review, the species of the genera *Podostroma* and *Monosporascus* would probably be great sources for producing macrocyclic trichothecenes. Moreover, we propose that the macrocyclic trichothecene roridin E could be formed via acylation or esterification of the long side chain linked with C-4 to the hydroxyl group at C-15, and vice versa. More assays and evidences are needed to support this hypothesis, which would promote the verification of the proposed pathway.

## 1. Introduction

**Figure 1:**
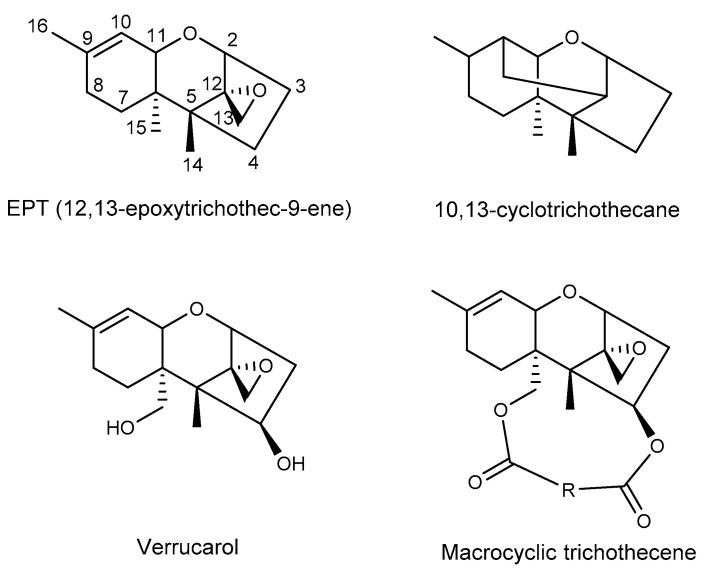
The tricyclic skeleton of trichothecene and macrocyclic trichothecene structure.

Trichothecenes are a family of sesquiterpenoid mycotoxins produced by multiple genera of fungi, including plant and insect pathogens, and they are of great concern because they are toxic to animals and humans and frequently detected in cereal crops, especially in wheat, barley, maize and oats [1,2,3,4]. In the European Union, 57% of the collected food samples have been contaminated with deoxynivalenol (DON) [5], and a high proportion of UK oats were believed to contain high concentrations of the trichothecenes T-2 and HT-2 according to a survey of commercial crops [6]. These toxins consist of over 200 structurally distinct molecules, all of which are characterized by a three-ring molecule known as 12,13-epoxytrichothec-9-ene (EPT; Figure 1) [7,8]. One obvious structural variation can divide trichothecenes into two groups: the simple trichothecenes and the macrocyclic ones (also called Type D trichothecenes, Figure 1) [9,10]. The macrocyclic trichothecenes possess an additional ring at C-4 and C-15 of EPT, formed by esterification of the hydroxyls at relevant positions with a 12- or 14-carbon chain. On the contrary, the simple trichothecenes in the other group do not have a macrolide ring.

The biochemistry, bioactivity and biosynthesis of simple trichothecenes have been studied extensively [11,12]. In *Fusarium*, the biosynthetic pathway of the representative trichothecenes, e.g., DON, nivalenol and T-2 toxin, have been fully elucidated [11,12,13]. All trichothecenes share the same starting unit, initiated by cyclization of the primary metabolite farnesyl diphosphate to form trichodiene, which was catalyzed by the terpene cyclase trichodiene synthase (Tri5). Subsequently, trichodiene undergoes a series of oxygenations (Tri1, Tri4, Tri11 or Tri13), acylations (Tri3, Tri7, Tri16 or Tri101) and sometimes other modifications to create the simple trichothecene analogs [10,12]. Most genes responsible for these catalytic processes have been identified individually, and they are often located next to one another in the trichothecene biosynthetic gene (*TRI*) cluster [14,15,16,17]. However, the same gene homologs from different genera sometimes have diverse functions [10,18]. For instance, in *Myrothecium* sp. and *Trichoderma* sp., the *tri4*-encoded cytochrome P450 monooxygenase catalyzes oxygenation of trichodiene at three carbons (C-2, C-11, and C-13), but Tri4 of *Fusarium* catalyzes four carbons of trichodiene (combined with C-3) [19,20,21]. Proctor et al. declared that the ability of Tri4 to catalyze three oxygenations is more ancestral than the ability to catalyze four reactions [10]. Likewise, Tri8 exhibits two mutually exclusive deacetylation at C-3 or C-15 among different *Fusarium* species [22,23,24]. The numbers and arrangement of *TRI* genes per cluster also vary in different fungi [10,18,25]. The *TRI* cluster of *Fusarium sporotrichioides* contains 12 genes related to trichothecene biosynthesis, but the cluster of *Trichoderma arundinaceum* just contains 5 genes [10]. The other biosynthetic genes of *T. arundinaceum*, e.g., *tri5* initiating biosynthesis of trichothecene, are located in other loci [18]. In *Myrothecium* and *Stachybotrys* species, the *TRI* genes at other loci were paralogs of genes in the main cluster [10].

The genetic bases for most simple trichothecenes have been elucidated by functional analyses of *TRI* genes; however, the genetic bases for macrocyclic trichothecenes, namely Type D trichothecenes, are not completely elucidated, especially about the formation of the macrocycle, which is composed of polyketide- and isoprenoid-derived moieties [9,26,27]. Genes *tri17* and *tri18* were presumed to be involved in macrocycle formation [7,26]. The objective of this review is to summarize the latest developments on macrocyclic trichothecenes, including newly discovered compounds, bioactivities, hypothetic functions of *tri17* and *tri18*, and putative biosynthetic pathway. This review provides a new insight into the biosynthetic mechanism of macrocycle in macrocyclic trichothecene.

## 2. Macrocyclic Trichothecenes and the Producing Strains

Trichothecenes can be isolated from many fungal genera, such as *Fusarium*, *Isaria*, *Microcyclospora*, *Myrothecium*, *Peltaster*, *Spicellum*, *Stachybotrys*, *Trichoderma*, *Trichothecium*, *Cephalosporium*, *Cylindrocarpon*, *Memnoniella*, *Phomopsis*, *Verticimonosporium* etc. [7,10,28]. Macrocyclic trichothecenes are produced by the genera *Myrothecium* [29,30,31], *Podostroma* [32], *Stachybotrys* [33], *Calcarisporium* [34], *Cercophora* [35], *Cylindrocarpon* [36], *Dendrodochium* [37], *Phomopsis* [38], and *Verticinimonosporium* [39]. It is worth noting that *Baccharis*, belonging to the *Asteraceae* family, is the only plant genus that can produce trichothecenes [40]. The macrocyclic trichothecenes can be further classified as verrucarins (mainly C27 compounds), or roridins and satratoxins (mainly C29 compounds) according to the carbon number of side chain [28,41,42]. The side chain with 12- or 14-carbon atoms was esterified to the tricyclic skeleton via hydroxyl groups at C-4 and C-15 of EPT [10].

**Figure 2:**
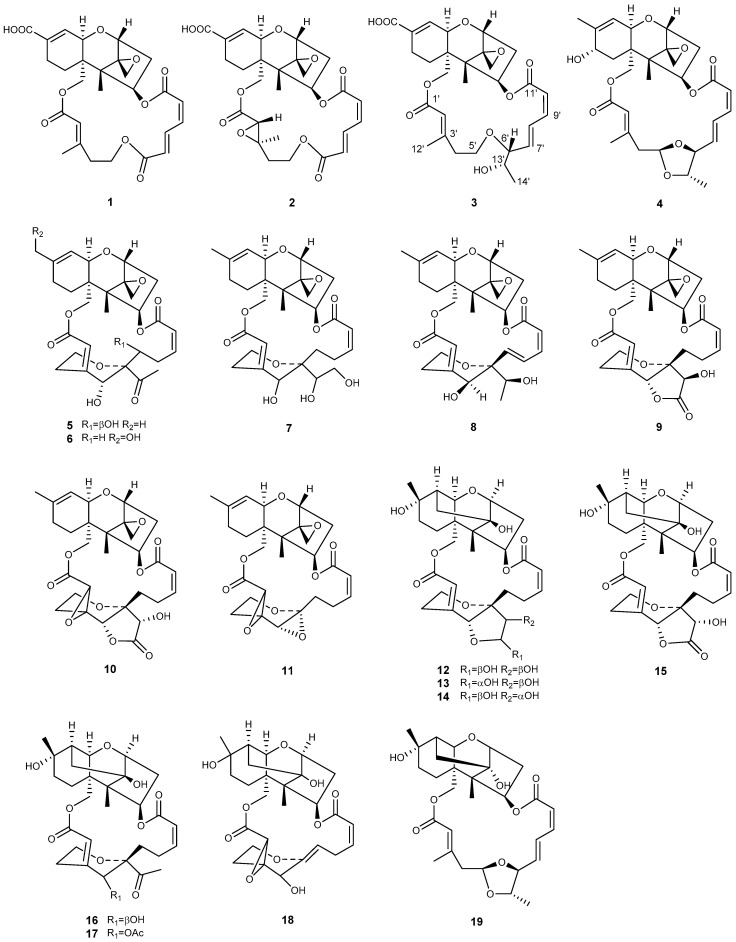
Structures of compounds **1**–**19** isolated from *Myrothecium*.

**Figure 3:**
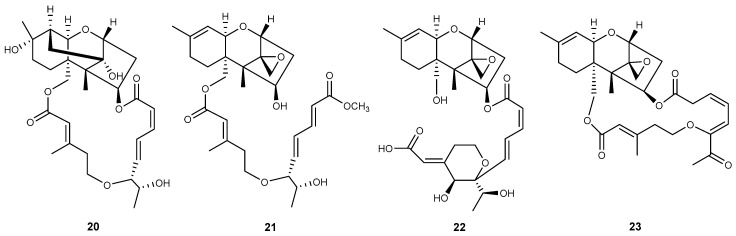
Structures of compounds isolated from *Podostroma* (**20**–**22**) or *Stachybotrys* (**23**).

New macrocyclic trichothecenes are constantly being discovered. Here, the novel macrocyclic trichothecenes isolated over the past decade are reviewed. There were 23 compounds related to macrocyclic trichothecene newly discovered between 2010 and 2019, and most of them were isolated from *Myrothecium* (Table 1; Figure 2). Only three compounds were isolated from *Podostroma*, and one from *Stachybotrys* (Figure 3). Four compounds belong to verrucarin-like trichothecenes (**1**, **2**, **11**, **18**), and the others with a side chain of 14-carbon atoms could be recognized as roridins or satratoxins. It was reported that roridin may be the precursor of the respective verrucarin, because the C_2_-side chain at C-6′ of roridin might be cleaved by oxidation to form verrucarin [41,43]. Compounds **1**–**11** and **23** have a standard tricyclic core, namely EPT; however, compounds **12**–**20** lack the epoxide at C-12, C-13. Verrucarin Y (**1**) and verrucarin Z (**2**) were isolated from *M. roridum* M10 by Muhammad et al. [42]. Verrucarin Y (**1**) shares the same structure with epiroridin acid (**3**) [29], except that the latter possesses an additional C_2_-side chain at C-6′, implying that epiroridin acid (**3**) may be the homologue of verrucarin Y (**1**) [41]. Verrucarin Z (**2**) has an epoxide ring at C-2′, C-3′ instead of the olefinic bond of verrucarin Y (**1**) at the same place, and the number of epoxide rings always has a significant impact on their biological activities [42]. Several macrocyclic trichothecenes with additional rings have also been isolated from *Myrothecium* (compounds **5**–**18**), such as mytoxin, satratoxin, vertisporin, roritoxin, and myrothecine, the names of which generally indicated the genus that this kind of toxins were firstly isolated from [41]. The second ring is closed by the connecting between C-6′ and C-12′ of the side chain. A third ring exists in some compounds, which is formed via an ester linkage between C-12′ and C-14′ (**9**, **10**, **15**) [30,44]. Roritoxin E (**10**) [30], 6′,12′-epoxymyrotoxin A (**11**) [45], and 2′,3′-epoxymyrothecine A (**18**) [46] have the epoxide ring at C-2′, C-3′; furthermore, 6′,12′-epoxymyrotoxin A (**11**) has an additional epoxide ring at C-6′, C-12′. Due to the absence of the epoxide at C-12, C-13, compounds **12**–**20** belong to 10,13-cyclotrichothecane derivatives [44,47]. Generally, 10,13-cyclotrichothecane macrolides have been proved to be less cytotoxic than the trichothecenes with the epoxide at C-12, C-13 [46,47,48]. Roridin F (**21**) and satratoxin I (**22**) discovered from *Podostroma cornu-damae*, a deadly poisonous mushroom, are not complete macrolides, because the macrocyclic ester bridge between C-4 and C-15 are not closed; however, they might be transformed into roridin E and satratoxin H, respectively, through a one-step esterification [32]. Chartarene D (**23**) is the only macrocyclic trichothecene isolated from *Stachybotrys* during the past decade [33]. This could be because the *Stachybotrys* fungi are not great source of trichothecenes, and most of them have been discovered before [28]. Search in the database Web of Science about macrocyclic trichothecenes reported during the last twenty years, implies that there is a possibility that more novel macrocyclic trichothecenes will be isolated from *Myrothecium* and *Podostroma*, especially the latter.

## 3. Biological Activities of Macrocyclic Trichothecenes

Macrocyclic trichothecenes have been shown to possess diverse biological activities, such as antibiotic [51], antifungal [41], antimalarial [52], antiviral [53], and anticancer activities [54,55]. In this review, the biological activities of macrocyclic trichothecenes against cancer cell lines were collected and compared (Table 2). The cytotoxicities against the identical cancer cell line varied notably between different compounds, which could explain the structure-activity relationship of macrocyclic trichothecenes.

Firstly, the existences of a double bond at C-9, C-10 and an epoxide at C-12, C-13 are the most important structural features, which contributes to the significant differences in biological activity (Table 2) [1,45,46,56]. The compounds lacking this epoxide and a double bond at C-9, such as compounds **12**–**20**, are not likely to show cytotoxicity with a nanomolar level. Secondly, an increase of the additional epoxide groups on the macrolide ring remarkably promote toxicity [1,57]. The 6′,12′-epoxymyrotoxin A (**11**), bearing an epoxide ring at C-6′, C-12′, showed quite strong cytotoxicity towards the KB and NCI-H187 cancer cell lines [45]. The substituent groups of other sites also affect the bioactivity. The carboxylation at C-16 could significantly reduce the cytotoxicity of epiroridin acid (**3**) compared to epiroridin [29]. The 12′-O-acetyl group in myrothecine G (**17**) enhanced bioactivity [44]. However, the hydroxylation of C-12′ decreased roridin E toxicity more than 1000-fold [58]. On the contrary, it has been reported that roridin H 8α-hydroxylation increased cytotoxicities towards MCF-7, Hela cells KB3.1, and skin cancer cells A431, but the acetylation of this hydroxy group or no oxidation at C-8 decreased activity towards all the mentioned cell lines [41]. Compounds **6**, **9**, **14**–**17** were evaluated for their in vitro cytotoxicities against K562 and SW1116, and the relatively large differences in IC_50_ values suggested that the slight changes of the substituent group have obvious effects on the biological selectivity of macrocyclic trichothecenes against cancer cell lines [44]. There have been some studies suggesting that the breakage of the macrocyclic ring could dramatically decrease the cytotoxicity [45,54], and it was supported by the fact that roridin F (**21**) and satratoxin I (**22**) without macrocyclic ring were almost inactive against cancer cell lines (data not included in Table 2) [32]. All these data indicated that even small alterations in the molecular structure can lead to a substantial change in biological activity or selectivity against cancer cell lines [41,59]. It should be noted that considerable research have pointed out that the macrocyclic trichothecenes have more potential than the other types of trichothecenes to become antitumor agents [41,60]. Therefore, more attention should be paid to exploration of the structure-activity relationships in the future.

There are some macrocyclic trichothecenes displaying other biological activities, such as antifungal and antimalarial activities. Verrucarin Z (**2**) showed a similar activity against *Mucor miehei* at a concentration of 50 μg/disk when compared with the positive drug nystatin [42]. The 8a-hydroxyroridin H (**4**) and myrothecin A (**19**) exhibited bioactivities against plant pathogenic fungi *Rhizoctonia solani* and *Fusarium oxysporum* [49]. Manami et al. suggested that the C-12-epoxide of trichothecene was essential for the antifungal activity against *Cochliobolus miyabeanus* [61]. Besides the cytotoxicity against the KB and NCI-H187 cell lines, 7′-hydroxymytoxin B (**5**) and 6′,12′-epoxymyrotoxin A (**11**) exhibited strong antimalarial activity against *Plasmodium falciparum* with 86.1% (1.84 nM) and 98.85% (2 nM) parasite inhibition, respectively [45].

## 4. Biosynthetic Pathway

**Figure 4:**
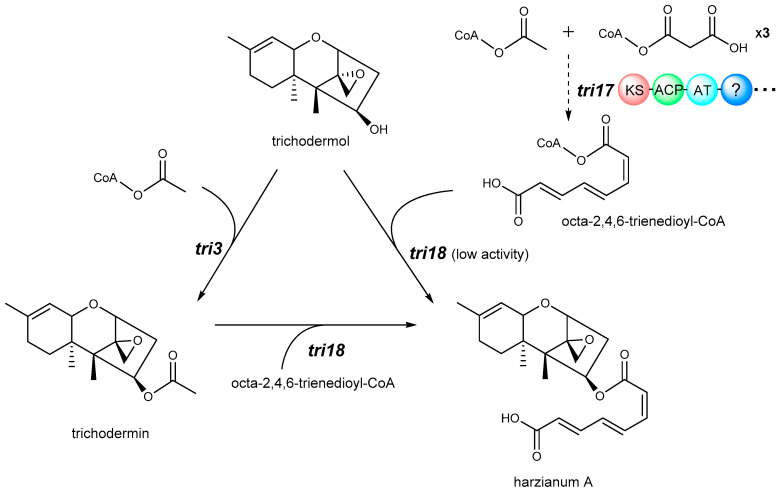
Biosynthetic pathway of harzianum A from trichodermol in *Trichoderma arundinaceum*. Genes are indicated in black italics.

Despite considerable published research on macrocyclic trichothecene, the biosynthetic pathway of Type D trichothecene remains obscure, especially on how the macrocycle is formed and linked to EPT. To date it has been well known that this mechanism is associated with genes *tri17* and *tri18*, but how they function is still being studied (Figure 4). According to previously reported BLAST analyses and function prediction [10,62], *tri17* is regarded as a polyketide synthase gene, and *tri18* as an acyltransferase gene. Based on the existence of *tri17* and *tri*18 in the *Stachybotrys TRI* cluster, Semeiks et al. speculated that the polyketide portion catalyzed by Tri17 protein participate in the biosynthesis of macrocyclic trichothecene in *Stachybotrys* [26]. Through gene deletion and complementation analyses, gene *tri17* in *Trichoderma arundinaceum* IBT 40,837 (Ta37) was confirmed to be essential for synthesis of the polyketide side chain of harzianum A (Figure 4). Furthermore, the complementation of *M. roridum tri17* into Ta37 could recover harzianum A production, demonstrating that *tri17* from different species played a similar role in biosynthesis of the polyketide-derived substituents of macrocyclic trichothecene [10]. Polyketide synthase is generally a delicate and complicate enzyme, containing several functionally diverse domains, including acyl carrier protein (ACP), acyl transferase (AT), ketosynthase (KS), ketoreductase (KR), dehydratase (DH), and enoylreductase (ER), all of which conjointly and sequentially function to generate a long branched-chain polyketide [63,64]. The polyketide synthases for other polyketide-derived compounds, such as tylosin [65], erythromycin A [63], and nanchangmycin [66], have been studied for many years. However, as a newly identified polyketide synthase gene, *tri17* has not been investigated thoroughly, and the functions of different domains have not been determined yet.

On the other hand, the only identification work for *tri18* was presented by Laura et al. in 2019 [62]. They proposed that the conversion of the intermediate trichodermol to harzianum A in Ta37 needed two acyltransferases, Tri3 and Tri18. Tri3 catalyzes 4-O-acetylation of trichodermol to give trichodermin, then Tri18 catalyzes the replacement of the resulting C-4 acetyl group with octa-2,4,6-trienedioyl (Figure 4). With deletion of gene *tri18* in Ta37, *tri18* mutants did not produce detectable levels of harzianum A, but the mutant genes increased trichodermol production, indicating that Tri18 accounts for the esterification of octa-2,4,6-trienedioyl to the C-4 of trichothecene. Moreover, Tri18 was presumed to be capable of transforming trichodermol to harzianum A directly, but the catalytic activity was quite low. This hypothesis requires more functional identification experiments in vitro to provide evidences.

**Figure 5:**
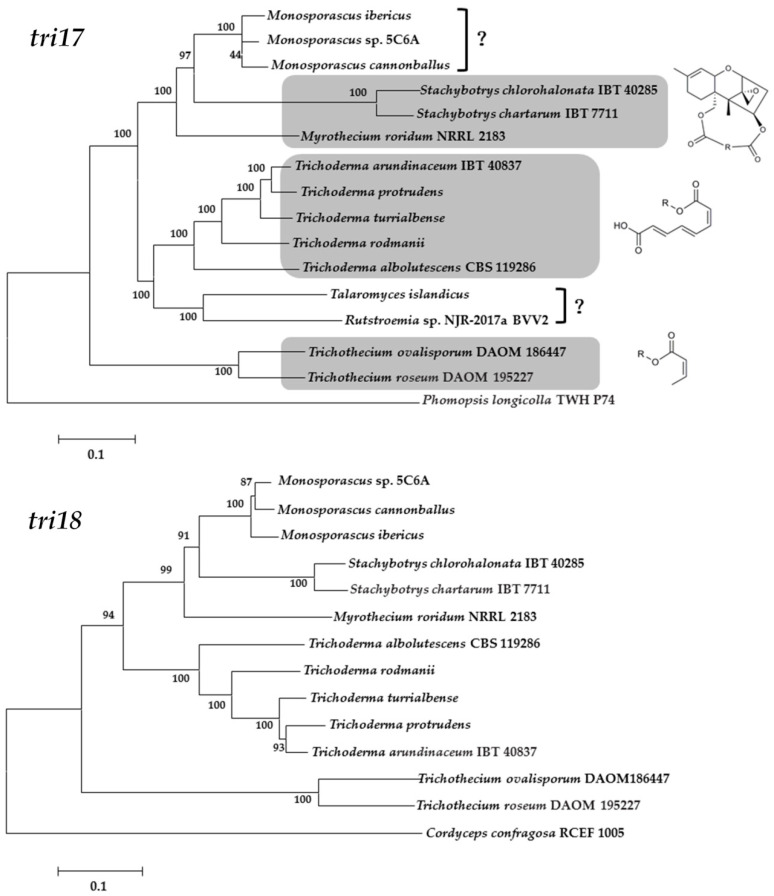
Maximum likelihood trees inferred from sequences of *tri17* (**top**) and *tri18* (**bottom**) and related homologs according to NCBI BLAST algorithms. Numbers near branch nodes are bootstrap values based on 1000 pseudoreplicates.

To obtain insight into the distinction of gene homologs and the evolutionary relationship of the macrocyclic trichothecene-producing species, phylogenetic trees for *tri17* and *tri18* were generated in the present review (Figure 5). Some of these gene homologs came from the trichothecene-producing strain genomes, and some from the resulting sequences with high similarity according to NCBI BLAST algorithms (Table S1). There were still some species without any reports on their genome information, such as *Podostroma*, *Calcarisporium*, *Cercophora*, *Cylindrocarpon*, *Dendrodochium*, *Verticinimonosporium*, and *Baccharis*, therefore their genes were not included in phylogenetic trees. In the trees inferred from homologs of *tri17* and *tri18* genes, relationships among these species were generally consistent (Figure 5). Firstly, *Myrothecium*, *Monosporascus* and *Stachybotrys* formed a well-supported clade in both trees. *Monosporascus* is also one plant pathogen affecting muskmelon and watermelon, and there are just a few studies indicating that natural products of *Monosporascus* species included octocrylene, squalene, several hexaketide and pentaketide compounds [67,68]. Since *Myrothecium* and *Stachybotrys* are the predominant fungi producing macrocyclic trichothecenes, it is a reasonable hypothesis that *Monosporascus* could produce macrocyclic trichothecenes as well. Secondly, *Trichoderma* and *Trichothecium*, respectively, forms an independent clade, and *Trichoderma* is more close to the *Myrothecium*-*Stachybotrys* clade than *Trichothecium*. In fact, the representative trichothecene derived from *Trichoderma*, harzianum A, has a longer side chain at C-4 than the representative trichothecene of *Trichothecium*, trichothecin (octatrienoyl group vs. butenoyl group, Figure 5) [10]. Although *Trichoderma* and *Trichothecium* have *tri17* and *tri18* genes, there is no report to date showing that macrocyclic trichothecenes could be isolated from them. Since Tri17 may be responsible for formation of the octatrienoyl substituent or other polyketide-derived substituents, and Tri18 catalyzes acetylation of the consequent substituents to EPT, there is still a puzzle how the long chain polyketides of macrocyclic trichothecene were converted to a closed ring. Either the unique gene *tri18* from *Myrothecium* or *Stachybotrys* also participates in the process, or there is still an unknown gene contributing to closure. In addition, it should be noted that we did not find any homologs similar to *tri18* in the root species in *tri17* gene tree, *Phomopsis longicolla*, which was the only species of the genus with genome information in NCBI.

**Figure 6:**
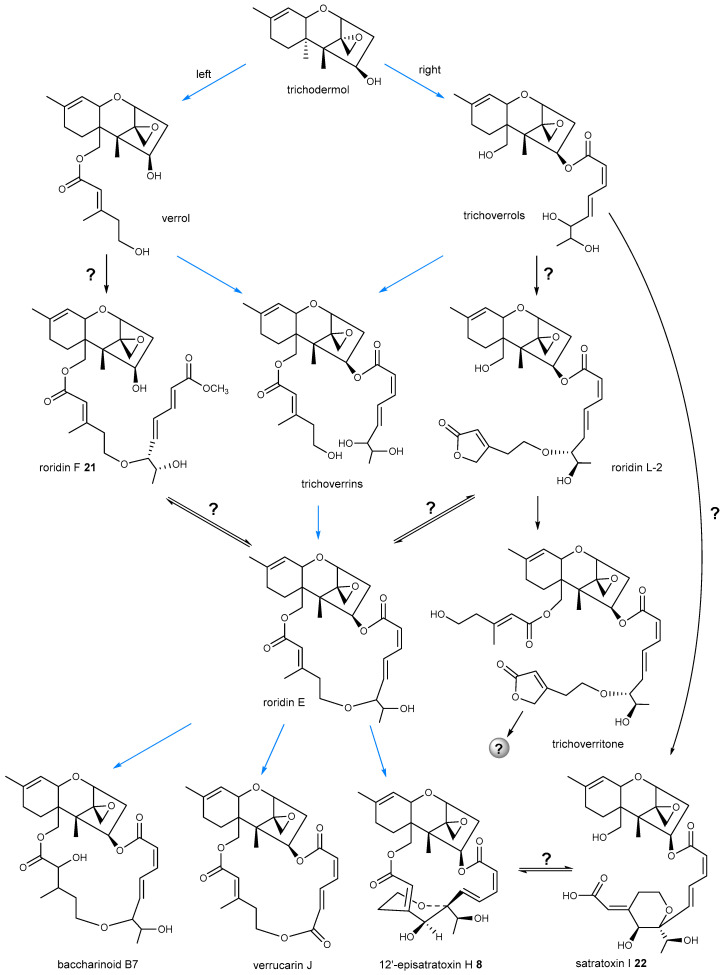
Plausible biogenetic pathway of macrocyclic trichothecenes. Blue arrows indicate the most predicted pathway; interrogation marks indicate that the process between two compounds still remains uncovered; gray ball represents a predicted unknown compound with a very large ring.

Although not all genes involved in the biosynthesis of macrocyclic trichothecene have been identified in detail, there are still a few studies proposing several possible biosynthetic pathways of macrocyclic trichothecenes [26,69,70,71]. The most commonly predicted biosynthetic pathway is shown as blue arrows in Figure 6. As initial compound of macrocyclic trichothecene, trichodermol can react with polyketide chains through esterification at two different sites: C-15 (left) or C-4 (right). A hydroxyl group at C-15 is a prerequisite for C-15 acetylation and subsequent esterification [10,72]. After oxidized, the 15-OH in trichodermol can be esterified with a 5-hydroxy-3-methylpent-2-enoic acid to form verrol, while the hydroxyl group at C-4 can be substituted by 6,7-dihydroxy-2,4-octadienoate to form trichoverrols [28]. If both the two hydroxyl groups are esterified consecutively, it will convert to trichoverrins. Subsequently, catalyzed by an unidentified protein, a dehydration between the two chains occurs to give the roridin E which constructs a macrocycle. Roridin E can be transformed to diverse derivatives by a series of slight modifications. As mentioned above, verrucarin J can be generated if the C_2_-side chain at C-6′ of roridin E is oxygenated [41,43]. Similarly, after being continuously oxygenated at C-12′, C-6′ and C-12′ can be linked to form **8** with a pyran ring [50]. The hypothesis could explain the generation of the most macrocyclic trichothecenes; however, the isolations of several compounds, some of which were recently discovered, required the hypothesis to be modified. Roridin F (**21**) reported in 2019 [32] and roridin L-2 [33,73] seem to be formed by cutting off the connection at C-4 or C-15 of roridin E. For roridin L-2, it also needs to undergo a cyclization of the hydroxyl group at C-1′ and C-12′. Nevertheless, there is a possibility that **21** could be generated by attaching a free 6,7-dihydroxy-2,4-octadienoate to the hydroxyl group at C-5′ of verrol. Then, **21** could be sequentially converted to roridin E through acylation or esterification. Similar processes may happen to roridin L-2 and satratoxin I (**22**) [32]. Compound **22** could be created through hydrolyzing at C-15 of **8**, or attaching a free 5-hydroxy-3-methylpent-2-enoic acid to the end of octadienoate fused to trichoverrols. Until now, there is no obvious evidence showing how **21**, **22** or roridin L-2 is formed, and whether the conversion between roridin E and **21** or roridin L-2, between **8** and **22** is reversible. The puzzle may be explained by identifying the specific undiscovered gene accounting for construction of the macrocycle, or knocking out gene *tri18* in *Myrothecium*, *Podostroma*, and *Stachybotrys*, all of which are able to produce macrocyclic trichothecenes. Since gene *tri18* in Ta37 accounted for esterification of octa-2,4,6-trienedioyl, deletion of it in macrocyclic trichothecene-producing strains may cut off the formation of macrocycle, resulting in an accumulation of verrol, or roridin F (**21**) and their derivatives. If final products do contain **21** and its derivatives, it suggests that **21** could be generated from verrol, meaning that there is a second putative pathway to form macrocyclic trichothecenes. In addition, isolated from *Myrothecium* [73] and *Podostroma* [32], trichoverritone was generated by esterifying a free 5-hydroxy-3-methylpent-2-enoic acid to the hydroxyl group at C-15 of roridin L-2. It remains unclear whether the side chains of trichoverritone could be cyclized; if it did, the consequent novel compound would possess a 20-membered ring.

## 5. Conclusions and Outlook

Trichothecenes have been studied for more than 40 years. Initially, trichothecenes were well known and studied as the important contamination sources of grains [2,74], building materials [75,76], and air-handling systems [7]. However, because of a large number of identification of new macrocyclic trichothecenes and their diverse bioactivities, they have drawn much attention from chemists and pharmacists as research hotspots [41,77,78]. We have summarized the macrocyclic trichothecenes reported during the last ten years and their biological activities. Although there are many species capable of macrocyclic trichothecene production, the newly discovered compounds were isolated from *Myrothecium*, *Podostroma* and *Stachybotrys*. Meanwhile, summary review in bioactivity studies revealed that the small changes between structurally similar compounds isolated from different species can result in a considerable alteration on bioactivity and/or selectivity against cancer cell lines [41,59], which again confirmed the great potential of macrocyclic trichothecenes as antitumor drugs. We also overviewed the recent advances in the identification of genes involved in the formation of macrocycle and plausible biosynthetic pathways. Knowledge obtained from functional analyses of *tri17* and *tri18* genes in *Trichoderma* has contributed significantly to insights into the evolutionary history of macrocyclic trichothecene biosynthesis. In terms of phylogenetic trees generated in the review and the presence of the highly similar genes *tri17* and *tri18* in *Monosporascus*, it was suggested that these species would probably be a source of macrocyclic trichothecenes. Furthermore, based on several newly isolated intermediates, we propose that there is a second biosynthetic pathway of macrocyclic trichothecene. An identification assay of the genes responsible for constructing the macrocycle in macrocyclic trichothecene-producing strains (maybe *tri18* or other unidentified gene), will be critical to more precisely clarify the biosynthesis mechanism of potential anticancer compounds.

## Figures and Tables

**Table 1:** Macrocyclic trichothecenes newly discovered over the last decade.

**Genera**	**No.**	**Compound**	**Ref.**
*Myrothecium*	**1**	verrucarin Y	[42]
	**2**	verrucarin Z	[42]
	**3**	epiroridin acid	[29]
	4	8a-hydroxyroridin H	[49]
	**5**	7′-hydroxymytoxin B	[45]
	**6**	16-hydroxymytoxin B	[44]
	**7**	13′,14′-hydroxymytoxin B	[46]
	**8**	12′-episatratoxin H	[50]
	**9**	14′-dehydrovertisporin	[44]
	**10**	roritoxin E	[30]
	11	6′,12′-epoxymyrotoxin A	[45]
	**12**	dihydromyrothecine C, 1a	[47]
	**13**	dihydromyrothecine C, 1b	[47]
	**14**	myrothecine D	[44]
	**15**	myrothecine E	[44]
	**16**	myrothecine F	[44]
	**17**	myrothecine G	[44]
	**18**	2′,3′-epoxymyrothecine A	[46]
	**19**	myrothecin A	[49]
*Podostroma*	**20**	miophytocen D	[32]
	**21**	roridin F	[32]
	**22**	satratoxin I	[32]
*Stachybotrys*	**23**	chartarene D	[33]

**Table 2:** Cytotoxicity of macrocyclic trichothecenes against diverse human cancer cell lines in vitro.

	**Cytotoxicity (IC_50_)**										
**Compound**	HepG-2 ^a^	MCF-7	SF-268	K562	SW1116	KB	NCI-H187	A549	SMMC-7721	SGC-7901	Ref.
**3**	0.380 ± 0.03 μM	0.170 ± 0.01 μM	0.751 ± 0.03 μM				0.360 ± 0.05 μM ^b^				[29]
**5**						2.81 nM	5.99 nM				[45]
**6**				2.87 μM	0.18 μM						[44]
**7**	49 ± 3.35 nM	63 ± 2.38 nM						53 ± 3.36 nM	46 ± 2.88 nM		[46]
**8**	2.27 μM					1.42 μM					[50]
**9**				56 nM	200 nM						[44]
**10**									18.89 μM	0.46 μM	[30]
**11**						0.63 nM	0.79 nM				[45]
**12**						44.48 μM					[47]
**14**				8.2 μM	0.57 μM						[44]
**15**				15.98 μM	11.61 μM						[44]
**16**				0.97 μM	10.62 μM						[44]
**17**				1.53 μM	4.25 μM						[44]
**18**	32.03 ± 2.94 μM	18.13 ± 3.89 μM						36.45 ± 2.79 μM	30.33 ± 9.71 μM		[46]

^a^ These cancer cell lines represent human hepatoma cell line (HepG-2), human breast adenocarcinoma cell line (MCF-7), human glioma cell line (SF-268), chronic myeloid leukemia cell line (K562), colorectal carcinoma cell line (SW1116), human nasopharyngeal carcinoma cell line (KB), human small cell lung cancer cell line (NCI-H187), human lung adenocarcinoma cell line (A549), human hepatocellular carcinoma cell line (SMMC-7721), and gastric carcinoma cell line (SGC-7901), respectively. ^b^ This datum was the cytotoxicity of compound **3** against NCI-H460 (human non-small cell lung cancer cell line).

## Supplementary Materials

The following are available online at https://www.mdpi.com/2072-6651/12/6/417/s1, Table S1: Information on fungal strains and genome sequences examined in the current study.
