# Supplementary material for: Recent Advances on Macrocyclic Trichothecenes, Their Bioactivities and Biosynthetic Pathway
Source: Toxins (Basel). 2020 Jun 23;12(6):417. doi: 10.3390/toxins12060417 (PMC7354583; doi:10.3390/toxins12060417)
Supplement: Supplementary file 1 [file toxins-12-00417-s001.pdf]

Supplementary Material: Recent Advances on Macrocyclic Trichothecenes, Their Bioactivities and Biosynthetic Pathway

Muzi Zhu, Youfei Cen, Wei Ye, Saini Li and Weimin Zhang

Table S1. Information on fungal strains and genome sequences examined in the current study.

	GenBank Accession No.	
**Strain**	***tri17***	***tri18***
Myrothecium roridum NRRL 2183	PXOD00000000	PXOD00000000
Trichothecium ovalisporum DAOM 186447	PXOC00000000	PXOC00000000
Trichothecium roseum DAOM 195227	PXNY00000000	PXNY00000000
Trichoderma arundinaceum IBT 40837	PXOA00000000	PXOA00000000
Trichoderma albolutescens CBS 119286	MN136192.1	MN136192.1
Trichoderma rodmanii	QED55501.1	QED55500.1
Trichoderma turrialbense	QED55509.1	QED55507.1
Trichoderma protrudens	QED55506.1	QED55504.1
Stachybotrys chartarum IBT 7711	KL648755.1	KL648755.1
Stachybotrys chlorohalonata IBT 40285	KL659704.1	KL659704.1
Monosporascus sp. 5C6A	QJOB01000065.1	QJOB01000065.1
Monosporascus ibericus	RYP11167.1	RYP11166.1
Monosporascus cannonballus	RYO75583.1	RYO75582.1
Cordyceps confragosa RCEF 1005	NA[a]	AZHF01000010.1
Phomopsis longicolla TWH P74	ASM80074v1	NA
Talaromyces islandicus	CVMT01000015.1	NA
Rutstroemia sp. NJR-2017a BVV2	NJPS01000075.1	NA

[a] Not available.
